# The Signaling of Cellular Senescence in Diabetic Nephropathy

**DOI:** 10.1155/2019/7495629

**Published:** 2019-10-03

**Authors:** Yabing Xiong, Lili Zhou

**Affiliations:** ^1^State Key Laboratory of Organ Failure Research, National Clinical Research Center of Kidney Disease, Division of Nephrology, Nanfang Hospital, Southern Medical University, Guangzhou, China; ^2^Guangzhou Regenerative Medicine and Health Guangdong Laboratory, Guangzhou, China

## Abstract

Diabetic nephropathy is the leading cause of chronic kidney disease (CKD) in western countries. Notably, it has a rapidly rising prevalence in China. The patients, commonly complicated with cardiovascular diseases and neurologic disorders, are at high risk to progress into end-stage renal disease (ESRD) and death. However, the pathogenic mechanisms of diabetic nephropathy have not been determined. Cellular senescence, which recently has gained broad attention, is thought to be an important player in the onset and development of diabetic nephropathy. In this issue, we generally review the mechanisms of cellular senescence in diabetic nephropathy, which involve telomere attrition, DNA damage, epigenetic alterations, mitochondrial dysfunction, loss of Klotho, Wnt/*β*-catenin signaling activation, persistent inflammation, and accumulation of uremic toxins. Moreover, we highlight the potential therapeutic targets of cellular senescence in diabetic nephropathy and provide important clues for clinical strategies.

## 1. Introduction

Diabetic nephropathy (DN) has been the leading cause of CKD and renal failure in developed countries. In the past two decades, the morbidity and mortality of DN have been rising rapidly in the worldwide population [1–4]. Along with the kidney injury, diabetic patients often suffer from multiple complications, such as retinopathy, neuropathy, and cardiovascular diseases. All of them contribute to a high risk of death [5]. Besides health problems in patients, DN also leads to a heavy burden to the society.

Previous reports indicate that the mechanisms of DN involve a multifactorial interaction of metabolic and hemodynamic factors such as high blood glucose, advanced glycation end-products (AGEs), and the renin-angiotensin system (RAS). They further link to the activation of protein kinase C- (PKC-) induced generation of reactive oxygen species (ROS) [6, 7], which further mediates the activation of downstream transcription factor nuclear factor kappa-light-chain enhancer of activated B cells (NF-*κ*B). Thus, the main treatments of DN refer to modulate glycemic and blood pressure through insulin and RAS inhibitors. However, they could only delay the progression of DN but not prevent or cure it. Patients suffering from DN still inevitably reach the stage of ESRD at an alarming rate in both developed and developing countries [8–10]. Hence, the new pathogenic mechanisms except hyperglycemia and hypertension should be determined for a better management of DN. Recently, the emerging role of cellular senescence in DN has attracted a broad attention. However, a comprehensive elucidation has not yet been achieved. In the present review, we will focus on the role of cellular senescence and its related mechanisms in DN. Furthermore, we will explore the potential therapeutic targets of cellular senescence and provide important clues for clinical strategies in the management of DN.

## 2. DN and Renal Aging

The aging kidneys undergo a wide range of macrostructural changes, such as decreased cortical volume, increased surface roughness, and augmented numbers and sizes of cysts [11], which correspond to the typical microstructural features of glomerulosclerosis, tubular atrophy, interstitial fibrosis, and nephron loss [12]. Cellular aging or cellular senescence is the critical factor for the process of aging. Although the senescent cells remain viable, they show typical changes with enlarged and flattened cell bodies, apoptosis resistance, increased activity of senescence-associated *β*-galactosidase (SA-*β*-gal), and upregulation of cyclin-dependent kinase (CDK) inhibitors including p16^INK4A^, ARF proteins, and p21 [13–16]. Furthermore, senescent cells, with the secretory features known as the senescence-associated secretory phenotype (SASP), could produce proinflammatory cytokines, such as tumor necrosis factor-*α* (TNF-*α*), interleukin-6 (IL-6), and monocyte chemoattractant protein1 (MCP-1), to greatly affect the neighboring cells [17, 18].

Recent reports show that CKD presents as a clinical model of premature aging. Wang et al. introduced a new concept of CKD-associated secretory phenotype (CASP), which indicates that senescent renal cells could secrete SASP components of various cytokines such as IL-1, IL-6, and TNF-*α* [19, 20]. Other reports also show that DN is highly associated with accelerated aging in various types of cells such as tubular cells, podocytes, mesangial cells, and endothelial cells [21–23]. Notably, hyperglycemia could directly induce cellular senescence in mesangial [24] and tubular cells [13, 25, 26]. Interestingly, high glucose could also induce macrophages to secrete SASP components, thus promoting the development of a low-grade inflammatory state and cellular senescence [20].

Besides hyperglycemia, the production of AGEs and induction of oxidative stress, chronic persistent inflammation, glucose toxicity, and lipid metabolism disorder under DN disease conditions could cooperatively promote the growing microenvironment for senescent cells [27]. Conversely, senescent cells could accelerate the progression of disease. The studies [28] show a strong association between glomerular expression of p16^INK4A^ and proteinuria. In addition, the excessive SA-*β*-gal activity and expression of p16^INK4A^ in tubules are positively correlated with interstitial fibrosis, tubular cell atrophy lesions. Of note, tubular cell senescence is intimately associated with BMI and blood glucose level, implicating that controlling cellular senescence plays a critical role in the therapeutics of DN.

**Figure 1:**
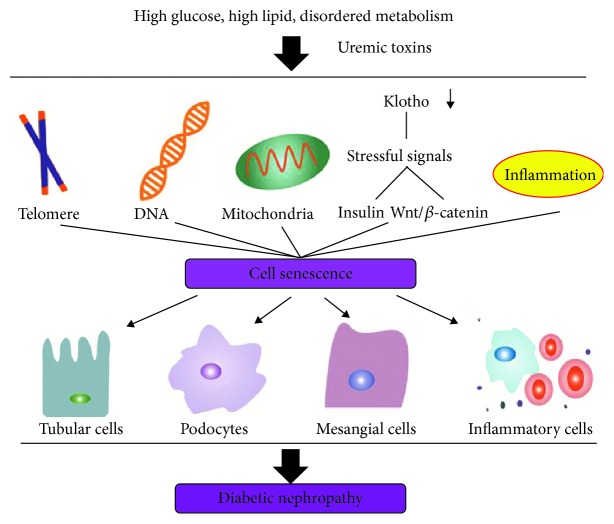
The cellular senescence in diabetic nephropathy.

There are two main consequences from the accumulation of senescent cells. First, as one might expect, because of permanent cell cycle arrest, cellular senescence may cause a loss of self-repair capacity and regenerative ability [29–32]. These would lead to the exhaustion of renal cells as well as other progenitor or stem cells. A study shows that the number of endothelial progenitor cells is 30%–50% lower in patients with chronic kidney disease than that in healthy subjects [33]. There are a limited reservoir, decreased population, and low renewal efficiency of stem cells in DN-affected kidneys [34, 35], which would certainly accelerate the progression of disease. Second, senescent cells could produce proinflammatory and matrix-synthesizing cytokines, such as IL-6 and TGF-*β*. These SASP-associated molecules may cause persistent inflammation and fibrosis, as well as stem and renal cell renewal dysfunction in a paracrine and autocrine fashion [17, 36]. Collectively, cellular senescence participates in many pathological processes to accelerate the progression of DN. In this review, we discuss the role of cellular senescence in the pathogenesis of DN, highlight new findings on the mechanisms of cellular senescence (Figure 1), and propose the novel strategies to treat patients with DN by targeting cellular senescence.

## 3. The Mechanisms of Cellular Senescence in Diabetic Nephropathy

### 3.1. Telomere Attrition and Cellular Senescence

Telomeres are stretches of repetitive DNA, which are located at the end of each chromosome. Telomeres protect the chromosomes from degradation or fusion [37, 38]. In repeated cell division, the length of a telomere may gradually shorten due to the lack of telomerase activity, an enzyme that helps to maintain the length of the chromosome.

Telomere shortening could occur in both type 1 and type 2 diabetes [39]. It can be accelerated by inflammation [40, 41], hyperglycemia, AGEs [42], and chronic oxidative stress [43], the main mechanisms of diabetes. Telomere attrition could trigger stress-induced premature senescence (SIPS). As early as 1999, it was found that telomere shortening is displayed in kidney diseases [44]. Consistently, recent studies show that high glucose induces accelerated senescence in proximal tubular cells, which is related to telomere shortening [28]. In both type 1 and type 2 diabetes, chromosomal telomere attrition is associated with renal cell senescence, proteinuria, and the progression of DN [45, 46]. After the prevention of telomere attrition, fenofibrate provides beneficial effects on the treatment of DN [39, 47]. The p53-p21-Rb signaling pathway is involved in the cellular senescence caused by telomere attrition under the condition of high glucose [48]. Besides the kidney parenchymal cells, the telomere of white blood cells in DN patients also displays the state of shortening. Although suggested as a biomarker in coronary heart disease [49], telomere shortening could also be applied in the diagnosis of DN. Supporting findings show that there is a high association between telomere length shortening and the progression of nephropathy through the mass population surveys [50].

### 3.2. DNA Damage and Cellular Senescence

DNA damage has been thought to be a main cause of cellular senescence since the late 1950s [51]. Besides the “wear and tear damage” during normal aging, there are various stress factors that could directly induce the damage of DNA, such as oxidative stress, ultraviolet (UV) or gamma irradiation [52, 53], chemotherapeutics [54], and hyperproliferation caused by the Ras oncogene [55]. Under diabetic conditions, hyperglycemia-induced generation of ROS and accumulation of AGEs may induce DNA damage and then trigger premature senescence in cells [56]. In type 1 diabetes rat models, AGEs and oxidative stress could induce DNA damage in both glomerular and tubular cells [57].

All of these DNA-damaged stressors could trigger a permanent DNA damage response (DDR) that leads to the activation of phosphatidylinositol 3 kinase-like kinases, such as ataxia telangiectasia-mutated (ATM) or ataxia telangiectasia-mutated and Rad3-related (ATR) kinases. These kinases subsequently activate P53 and its transcriptional target p21^CIP1/WAF1^. In turn, p21^CIP1/WAF1^ inhibits cyclin-dependent kinase 2- (CDK2-) mediated phosphorylation of the retinoblastoma protein (Rb). The hypophosphorylated Rb binds to the E2F transcription factor, preventing E2F through interacting with the transcription machinery. This process ultimately results in permanent cell cycle arrest, i.e., cellular senescence. This pathway is also called ATM/ATR-p21axis [23, 58]. Ultimately, senescent cells exhibit elevated levels of DNA damage response proteins 53BP1 and *γ*H2AX and poor repair capacity for DNA strands. These could induce the restructure of the epigenome (see below), such as CpG methylation patterns [59].

### 3.3. Epigenetic Alterations and Cellular Senescence

Epigenetics refers to heritable alterations in gene expression and phenotype without involving changes in the DNA sequence. Epigenetic modifications include cytosine methylation of DNA (DNA methylation, DNAme), histone posttranslational modifications (PTMs), and noncoding RNAs [60–63]. Alterations in the epigenome underlie the dynamic switching of chromatin between a transcriptionally silent compact structure (heterochromatin) and an active relaxed structure (euchromatin) that cooperatively regulate gene expression [64].

DNAme occurs primarily, but not exclusively, at the sites of cytosine-guanine (CpG) dinucleotides. DNAme usually represses gene transcription through recruiting repressor complexes or precluding transcription factors [65]. However, the regulatory effects of DNAme can vary from gene repression to gene activation, which largely depends on the genomic contexts of the targeted sequences, such as promoters, gene bodies, enhancers, and repeated sequences [62]. PTMs mainly include histone lysine acetylation (HKAc) and histone lysine methylation (HKme). HKAc activates gene expression, while HKme activates or represses transcription depending on the lysine residue modified and the extent of methylation (mono-, di-, or trimethylation) [64, 65]. Other histone modifications consist of phosphorylation, sumoylation, ubiquitination, ADP-ribosylation, and O-GlcNAcylation [66]. They are also important but not well-studied.

Epigenetic changes could appear in the very early stage of DN. In 5-week-old db/db mice, the expression of Agt, an important component of the renin-angiotensin system, is upregulated by histone H3K9 acetylation [67, 68]. Similarly, the hypomethylation of CpG islands in Claudin-1 gene [67, 68] is also found at a very early stage in diabetes before the increase in albuminuria.

Increasingly, epigenetic changes are thought to be important in the development of DN through the induction of oxidative stress. In a rat model of diabetes, H3K27me at the enhancer site of zeste 2 repressive complex 2 subunit (EZH2) dampens the expression of the endogenous antioxidant inhibitor thioredoxin-interacting protein (TXNIP) via repressing the transcription factor PAX6. The inhibition of EZH2 augments proteinuria, podocytopathy, and renal oxidative stress, through the inhibition of glomerular TXNIP expression [69]. In addition, high glucose-mediated TXNIP expression is coordinated by histone acetylation and methylation in diabetic kidneys [70]. Similarly, in podocytes, high glucose could induce CpG promoter hypomethylation and histone H3 hyperacetylation through the activation of histone GCN5 acetyltransferase, which would drive the expression of p66Shc [71, 72], the key regulator of oxidative stress and player in lifespan shortening [73]. Furthermore, high glucose could also recruit Suv420H2 methyltransferase and LSD1 histone dimethyltransferase to the gene promoter of superoxide dismutase 2 (SOD2), resulting in the downregulation of SOD2, a strong endogenous antioxidant [74].

Epigenetic alterations also play important roles in persistent inflammation and autophagy deficiency, two main mechanisms of cellular senescence. In vascular endothelial cells [75–77] and inflammatory cells, high glucose induces H3K4me1 activation through SET7 lysine methyltransferase to encode the proinflammatory factor NF-*κ*B [78]. More and more studies show the important roles of noncoding RNAs in the development of DN. In db/db mice, miRNA-125b represses H3k9me3 through the downregulation of Suv39h1 histone methyltransferase, which leads to the upregulation of MCP-1 and IL-6, the two chemoattractants for the differentiation and migration of monocytes and lymphocytes [79]. Another noncoding RNA named miRNA-146a, a well-known anti-inflammatory microRNA, increases in the early stage of DN to protect against inflammation and renal fibrosis through inhibiting the activation of M1 macrophage [80]. Additionally, high glucose induces the activation of histone deacetyltransferase 4 (HDAC4) in podocytes, which leads to the deacetylation and silence of STAT1, a named gene in promoting autophagy to exert renoprotective function [81]. Furthermore, the administration of histone deacetylase (HDAC) inhibitor trichostatin A reduces kidney injury through alleviating the loss of Klotho, a well-known antiaging protein [82]. These studies further confirm the mediating role of epigenetic alterations in cellular senescence.

### 3.4. Mitochondrial Dysfunction and Cellular Senescence

#### 3.4.1. Mitochondrial Reactive Oxygen Species (mtROS) and Mitochondrial Dysfunction

ROS can be both a trigger and an effector in the aging process and have been viewed as a cause of the aging process since 1996 [83]. Mitochondrial ROS (mtROS) could be driven by external stimuli, such as inflammatory cytokines, growth factors, or environmental toxins. The overproduction of mtROS increases in natural aging and age-related diseases [84]. The production of mtROS originates from the defects in the electron transport chain (ETC), which promotes electron leakage to form superoxide radicals, a key player in cellular senescence and accelerating aging [85]. The overproduced mtROS could damage protein, lipid, and DNA, which further trigger the pathological changes [86]. It is noteworthy that in diabetic nephropathy, the altered metabolic state requires extra demands of ATP, which would accelerate electron leakage to produce excessive ROS. The accumulative ROS in the mitochondrion has a detrimental effect in the integrity and content of mitochondrial DNA (mtDNA), which encodes the subunits of ETC complexes. This would further induce mitochondrial dysfunction and increase the production of ROS [87]. Notably, 30% of diabetic patients are suffering from mitochondrial dysfunction as well as reduced mitochondrial biogenesis, which is tightly associated with the severity of kidney diseases [88].

However, the exact role of ROS in cellular senescence is controversial. Although ROS is commonly considered as a cause of cellular senescence, recent findings show that it also extends Drosophila's lifespan, especially through respiratory complex I reverse electron transport [89]. Other reports also show that low levels of ROS could serve as second messengers to extent lifespan in *Caenorhabditis elegans* [90–92]. Moreover, chemical inhibition of glycolysis or exposure to metabolic poisons that block respiratory complex I (rotenone, paraquat, or piericidin A) or complex III (e.g., antimycin A) also prolong lifespan in *C. elegans* in a ROS-dependent manner [93]. Thus, the role of ROS homeostasis in cell aging under diabetic conditions may need more studies to confirm.

It is noteworthy that the production of mtROS could induce the mutations of mtDNA, which contributes to the aging process. This theory is confirmed by mitochondrial mutator mice, an early aging mice model expressing a proofreading-deficient DNA polymerase POLG*γ*. In those mice, the accumulated mutations of mtDNA significantly accelerate aging phenotype [94]. Recently, the evidences of mtDNA mutation are shown in DN patients. A study shows that mtDNA mutation G13997A in ND6 gene, a key gene encoding one of the subunits of respiration complex I (NADH dehydrogenase), positively correlates with the development of diabetes and related nephropathy [95]. However, some studies show that mutations of mtDNA are not major contributors to aging, especially in fruit flies [96]. The reason lies in that fruit flies are less sensitive to mtDNA mutations in adulthood than during development. Hence, more studies are needed to prove the correlation between mtDNA mutations and cellular senescence in renal cells.

#### 3.4.2. Mitophagy Impairment and Early Senescence

Mitophagy plays an important role in preserving healthy mitochondria via the removal of altered mitochondria, clearance of protein aggregates. Mitophagy exerts protective functions in inhibiting apoptosis, reducing ROS production, and anti-inflammation [97–99]. It is reported under diabetic condition, more than 50% of renal tubular cells exhibit fragmented mitochondria [100], concomitant with the significant upregulation of mtROS in the renal cortex [101]. These suggest the loss of mitophagy in DN.

Mitophagy depends on the signaling cascade of kinases. The most important kinase is the PTEN-induced putative kinase 1 (PINK1). Upon damage, PINK1 transduces signals to the cytosolic E3 ubiquitin ligase Parkin. Parkin then amplifies the signals of mitophagy by facilitating PINK1-mediated recruitment of optineurin (OPTN) and NDP52 [102, 103]. Optineurin (OPTN) contains an ubiquitin-binding domain with the ability of binding polyubiquitinated cargo and transporting cargo to form autophagosomes [104]. The loss of expression in Parkin correlates with lifespan shortening [105], while the overexpression of Parkin extends longevity [106]. Similarly, the knockdown of PINK1 shortens lifespan and accelerates aging [89]. Interestingly, recent report shows that OPTN is involved in high glucose-induced senescence in renal tubular epithelial cells [25].

A large body of studies show that mitophagy is defective in diabetic kidneys [100], concomitant with mitochondrial abnormalities, overproduced mitochondrial ROS, and reduced expression of PINK and Parkin [107]. Indeed, podocytes show a high rate of baseline autophagy with aging. However, under diabetic status *in vivo* and high glucose conditions *in vitro* [108], the high basal level of autophagy in podocytes is flawed, which facilitates cell injury, glomerular damage, and the progression of kidney diseases. Hence, it can be presumed that in a diabetic setting, defects in mitophagy could induce early senescence in different renal cells and further promote the progression of kidney diseases.

### 3.5. Falling Levels of Klotho and Dysfunction of the Klotho-FGF-23 Axis

Klotho is an antiaging protein and is predominantly expressed in normal tubular cells. Klotho could act on multiple signals such as insulin and Wnts [109–111] and exerts important protection in kidney function [112]. The decline of Klotho could be seen in an early stage of kidney diseases, and this deficiency is linked to accelerated aging, cellular senescence, vascular calcification, and oxidative stress [113]. However, the underlying mechanisms remain poorly understood. Some reports show Klotho protects against cellular senescence through activating the forkhead transcription factor FOXO, a negative regulator of mtROS generation [114]. Furthermore, Klotho also has endogenous anti-inflammatory effects [115, 116] and antifibrotic properties [109, 117, 118]. Klotho also plays a role in mineral metabolism disorders, which would further affect renal aging. Klotho promotes calcium absorption and phosphate excretion in kidneys [119] and serves as a permissive coreceptor of fibroblast growth factor 23 (FGF-23), the hormone regulating phosphate and vitamin D [120]. This is called the Klotho-FGF-23 axis. The loss of Klotho promotes hyperphosphatemia, a risk factor of senescence process [121] and longevity [122].

Diminished expression of Klotho is a common feature of DN and is observed at the earliest stage of the disease [123, 124]. The loss of Klotho could be associated with multiple mechanisms such as hypermethylation of Klotho gene [125, 126], NF-*κ*B-induced falling level of Klotho gene [127]. Recent studies show that increasing activity of integrin-linked kinase protein (ILK) reduces Klotho gene expression, which leads to cellular senescence in renal cells [128]. Large amounts of studies show that the loss of Klotho links to lifespan shortening, skin and muscle atrophy, osteoporosis, and calcification [129]. Under uremia condition, Klotho retards epithelial cell senescence through decreasing oxidative stress, NF-*κ*B activity, etc. [118]. A survey in humans further confirms that the serum level of Klotho declines with age, and Klotho gene displays single nucleotide polymorphism which correlates with reduced longevity and the pathophysiology of age-related disorders [111].

### 3.6. Wnt/*β*-Catenin and Cellular Senescence

Despite being relatively silent in normal adult kidneys, Wnt/*β*-catenin signaling is reactivated in a wide range of chronic kidney diseases (CKD), such as diabetic nephropathy, obstructive nephropathy, adriamycin nephropathy (ADR), polycystic kidney disease, and chronic allograft nephropathy [130–133]. The canonical Wnt/*β*-catenin signaling involves *β*-catenin dephosphorylation in serine/threonine residues, which leads to its translocation to the nucleus, where it binds to transcription factor T-cell factor (TCF)/lymphoid-enhancer binding factor (LEF) to induce the expression of downstream target genes [134–136]. However, in diabetic status, the accumulated intracellular ROS might divert the limited pool of *β*-catenin from TCF/LEF to forkhead box O- (FOXO-) mediated transcription [137, 138] that leads to insulin deregulation. Notably, the latter plays a pivotal role in the aging process.

Recent evidence suggests that the renin-angiotensin-aldosterone system (RAS) is mediated by Wnt/*β*-catenin signaling. Zhou et al. reported that the promoter regions of all RAS genes contain putative TCF/LEF-binding sites, and *β*-catenin induces the binding of LEF-1 to these sites in renal tubular cells. Ectopic *β*-catenin causes the upregulation of all RAS genes [139]. Notably, RAS activation contributes to renal aging through various mechanisms. Several studies discover that angiotensin II, a key substance of RAS, can induce senescence in renal cells and lead to the development and progression of age-related diseases [140–143]. Ang II induces premature senescence via both STAT3/mTOR-regulated autophagy and the p53/p21 pathway [140], which further drives fibrosis and redox state [144]. Notably, ROS can also induce the expression of p16^INK4A^, a trigger in cell cycle arrest and senescent phenotype, and activate TGF-*β*1 and NF-*κ*B signaling that could trigger inflammatory reaction in accelerated aging process [145]. Hence, RAS antagonism through administration of ACEI/ARB improves mitochondrial function and exerts antioxidative effects and displays age-retarding benefits [146].

Recently, Luo et al. reported that Wnt9a has a decisive role in driving tubular senescence and renal fibrosis, as well as evoking cell communication between senescent tubular cells and interstitial fibroblasts [147]. Although not studied in their studies, RAS activation is supposed to play a role in Wnt9a-induced cellular senescence. Supporting the presumption, several studies point out the benefits of inhibiting the RAS system in organ aging process. The related mechanisms involve the upregulation of prosurvival nicotinamide phosphoribosyltransferase gene (Nampt) and downregulation of p16^INK4A^ expression [148, 149], as well as the improvement of mitochondrial function [150–152].

### 3.7. Inflammation and Cellular Senescence

Chronic inflammation is a pathological feature of various CKD. Notably, inflammation serves as an important factor for accelerated aging. Unregulated inflammation has a key role in the pathogenesis and progression of autoimmune diseases such as presenile dementia, osteoporosis, and atherosclerosis [153]. Inflammation is also involved in the pathogenesis of obesity and diabetes and serves as an important mediator of aging [154, 155]. In addition, the senescence-associated decline of the adaptive immune system (immunosenescence) may further aggravate aging phenotypes at the systemic level because of the impaired immune surveillance [156, 157]. Moreover, senescent cells can secrete many proinflammatory factors, such as TNF-*α*, IL-6, PAI-1, and MCP-1, which may further aggravate inflammation [17, 157]. Recent studies show that AUF1, the mRNA decay factor, links inflammation and aging [158]. Deficiency of AUF1 induces marked cellular senescence and premature aging phenotype.

In diabetic nephropathy, the prominent inflammation is observed even in the beginning and ongoing stage of kidney injury. The upregulation of systemic and local renal inflammation occurs in the early stage of DN. In diabetic status, high glucose, AGEs, and oxidative stress could simultaneously induce the activation of NF-*κ*B, a known transcriptional signature of inflammation [18]. Through recruiting p300, NF-*κ*B triggers the activation of downstream effector iNOS, a promoter of oxidative stress and inflammation that causes extensive nitrotyrosine (NT) in proteins [159]. Genetic and pharmacological inhibition of NF-*κ*B signaling prevent age-associated diseases [160–162] including diabetic nephropathy [159] and natural aging [163, 164].

Additionally, Nod-like receptor 3 (NLRP3) inflammasome also plays a role in the development of inflammation under diabetic setting [165]. In response to diverse damage-associated molecular patterns (DAMPs) in aging, such as excess glucose, ceramides, amyloids, urate, and cholesterol crystals, NLRP3 inflammasome is intimately correlated with age-related diseases [166]. The NLRP3 inflammasome could activate caspase-1, which stimulates the maturation and secretion of IL-1*β* and IL-18 through cleaving their precursors [167]. Consequently, these proinflammatory cytokines accelerate the aging process through inhibiting autophagy [168]. Conversely, the impaired autophagy could trigger the accumulation of inflammasome to create a reciprocal activation loop [169]. Interestingly, inflammation could also interplay with many other mechanisms of cellular senescence such as telomere shortening, progressive DNA damage, oxidative stress, and altered epigenetics. These create an intricate network to induce cellular senescence in diabetic nephropathy.

### 3.8. Uremic Toxins and Cellular Senescence

Uremic toxins are endogenous waste products of metabolism. They are cleared predominantly by the kidneys. Three subgroups of uremic wastes are classified: (i) small water-soluble molecules (MW < 500 Da) such as urea, creatinine, and phosphate; (ii) middle (MW500~5000 Da) and large molecules (MW > 5000 Da) such as parathyroid hormone (PTH), IL-6, fibroblast growth factor 23 (FGF23), AGEs, advanced oxidation protein products (AOPPs), and other peptides; and (iii) small molecules (MW < 500 Da) but with high protein-binding abilities such as indoles, phenols, polyamine, and cresols [170]. Notably, the regular hemodialysis could only remove small molecules with the molecular weight lower than 500 Da. Furthermore, it is also difficult to remove protein-bound uremic toxins such as indoxyl sulfate (IS), p-cresyl sulfate (PCS), and 3-carboxy-4-methyl-5-propyl-2-furanpropionic acid (CMPF), due to their high protein-binding capability (90%) to plasma proteins [171, 172]. It is notable that the imbalance of gut microbiome in kidney diseases largely contributes to the formation and retention of uremic toxins [173], which would create a reciprocal activation loop that accelerates the progression of kidney diseases.

Uremic toxins could trigger senescence in various types of renal cells such as the proximal tubular cells [174, 175] and endothelial cells [176] through multiple mechanisms. The most commonly studied pathway is oxidative stress-induced NF-*κ*B signaling [177]. However, other mechanisms are also contributed. Some reports show that uremic wastes could induce mitochondrial dysfunction [178] and hypermethylation in Klotho gene [125], the two main mechanisms of cellular senescence in DN. As the well-known uremic toxins in DN, AGEs account for various mechanisms of cellular senescence. Although their detrimental role is firstly found in the longevity of C. elegans [179, 180], AGEs show the active role in promoting senescence phenotype in multiple organ systems including the kidneys in humans [181]. Studies show that AGEs trigger cellular senescence via oxidative stress-dependent p21 activation [182] and p16 expression [13, 183], inhibition of autophagy [184] through reducing PINK1/Parkin [185], and promotion of inflammation [186] in renal cells and others.

Taken together, the accumulation of uremic toxins would influence cellular senescence nearly in all aspects of mechanisms, which cooperatively and reciprocally promote and accelerate cellular senescence that contributes to the pathogenesis of DN.

## 4. Therapeutic Potentials

**Figure 2:**
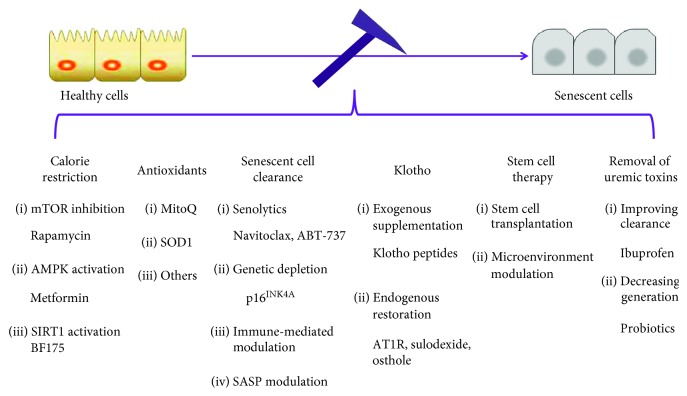
Therapeutic potentials against cellular senescence in diabetic nephropathy.

The therapeutic methods for DN nowadays refer to anti-RAS therapy using ACE inhibitors (ACEIs) or angiotensin II receptor blockers (ARBs) and glucose control. However, anti-RAS therapy only displays limited efficacy, partly because of the compensatory upregulation of renin expression [187–189]. Due to the metabolic memory of prior exposure to hyperglycemia, a single control of glucose fails to prevent the progression of kidney disease [190, 191]. Notably, several theoretical approaches might be applicable to target the aging process in DN (Figure 2).

It is well known that calorie restriction (CR) reduces oxidative stress and proinflammatory injury. In addition, CR modulates mitochondrial activity and increases the autophagy activity, thereby extending health and lifespan [192–194]. It has been shown that CR protects against cellular senescence through decreasing the expression of p16^INK4A^. Consequently, renal fibrosis is alleviated [192]. Actually, CR inhibits cell senescence through various mechanisms. The most studied is the mammalian target of rapamycin (mTOR) signaling. CR could deactivate mTOR through activating AMP-activated protein kinase (AMPK) [195]. Several kinds of drugs targeting this pathway are effective in the retardation of aging and age-related disease. Rapamycin, the mTOR inhibitor, could protect cellular senescence [196] and phosphate-induced premature aging [197]. While AMPK activator metformin reduces ROS production in podocytes and prevents DN [145]. Interestingly, long-term treatment with rapamycin may improve the quality of mtDNA in aging mice [198]. However, long-term CR has a long way to achieve for clinical practice, especially in DN patients because of the limited daily nutrition. Another promising drug is sirtuin1 (SIRT1) agonist. As a NAD^+^-dependent deacetylase, SIRT1 plays an important role in the aging process [199] and age-related phenotypes such as DN [200]. SIRT1 could be induced by CR. The agonists of SIRT1 such as BF175 and resveratrol could greatly ameliorate the pathogenesis of diabetic kidney disease [201]. And BF175 also shows less renal toxicity, suggesting the good prospects.

Theoretically, antioxidants can mitigate ROS-induced damage, such as DNA mutations and protein modifications, as well as delay telomere shortening. However, the advantages of antioxidants are needed to be further clarified in aging-associated diseases [202–205]. Chlorogenic acid could attenuate oxidative stress and inflammation in diabetic nephropathy, possibly through modulating Nrf2/HO-1 and NF-*κ*B pathways. However, no direct evidences show its effects on aging retardation under the diabetic setting [206]. Oppositely, the administration of mitochondria-targeted antioxidant MitoQ could dramatically ameliorate renal tubular injury in a diabetic mouse model. MitoQ reverses mitophagy through increasing the expression of PINK1 and Parkin [107]. Another important study shows that Cu/Zn-superoxide dismutase 1 (SOD1), a strong endogenous antioxidant, has a new role in aging. Deficiency of SOD1 prolongs lifespan and retards the process of cellular senescence [207]. Overexpression of SOD1 attenuates high glucose-induced endothelial cell senescence [17]. These suggest that SOD1 may act as a promising therapeutic in DN through retarding aging. However, because of side-effects such as tumor genesis and difficulties to control the degree of antioxidation, the reasonable application should be closely noticed in therapy for patients.

Another strategy to retard cellular senescence is the removal of these cells [208] by senolytics, a new kind of drugs with the ability to slow the aging process. Many of these agents could induce the clearance of senescent cells through upregulation of antiapoptosis systems such as the BCL-2 family of proteins (BCL-2, BCL-XL, and BCL-W) [209, 210]. The new senolytic molecules navitoclax and ABT-737 could occupy the inhibitory binding grooves of BCL-2, BCL-XL, and BCL-W, which counteract their antiapoptotic functions and initiate apoptosis in senescent cells [211]. Furthermore, immune surveillance [212] and genetic deletion of senescence-associated factor p16^INK4a^ [213] could also help to enhance the removal of senescent cells.

SASP modulation has also been proposed as a way to slow the aging process. SASP blockade would be another attractive treatment option [214, 215]. However, the interventions of proinflammatory pathways such as NF-*κ*B, p38-MAPK, or MAPK-activated protein kinase 2 could also influence the communications among healthy cells [216]. Notably, a recent finding shows that bromodomain-containing protein 4 (BRD4) inhibitors could modulate SASP with high specificity in senescent cells without influencing healthy subjects [217]. In histones, BRD4 binds to acetylated lysines, which results in the opening of chromatin and the activation of SASP process [217]. BRD4 inhibitors JQ1 and iBET are recently studied and found to be prospective in therapeutic of aging [218].

Restoration of endogenous Klotho or supplementation with exogenous Klotho could be a substitute therapeutic strategy for antiaging. Klotho could block Wnt/*β*-catenin signaling and its targets such as the renin-angiotensin system (RAS) and modulate the homeostasis of blood phosphate and vitamin D [139, 219]. In the aging process, Klotho gene displays hypermethylation [126]. Notably, Ang II type 1 receptor antagonist (AT1R) losartan could alter the posttranslational modifications on histones [220], suggesting a protective role in Klotho gene stability. In addition, some drugs such as sulodexide and osthole may enhance the expression of Klotho to prevent the progression of DN [221]. Considering the side-effects and toxicity of these molecular compounds, the direct supplement of Klotho may be the best way to provide renoprotecion. However, the clinical application of direct Klotho supplement is very limited because Klotho protein is with large molecular weight and complex structure. It is still promising that scientists are developing the small molecular peptides from the binding sites of Klotho with Wnt3a and other Wnts [222], suggesting the prospects for future clinical application.

The latest topics of therapeutics in aging are stem cell therapy [223]. A study shows that the injection of plasma from young mice into the circulation of aged mice induces a more youthful state [224]. Furthermore, early-in-life genetic manipulations could preserve the proliferative ability of the gut stem cells, which leads to lifespan extension [225]. In kidney disease, the supplement of mesenchymal stem cells (MSC) could prompt functional recovery [226]. Even in aged stem cells, incubation of young blood could still ameliorate the functional deficits [227, 228], suggesting the important role of microenvironment in stem cell renewal. Other studies also show that human umbilical cord-derived mesenchymal stromal cells could ameliorate renal fibrosis and cellular senescence and interestingly increase the expression of Klotho [229]. Regenerative therapies by exogenous stem cell transplantation into damaged tissues could improve natural aging and stress-induced premature senescence (SIPS) [230]. However, the stem cell transplantation in humans is more challenging because of the unpredicted disadvantages such as tumor genesis and needs to be demonstrated repeatedly.

Removal of uremic toxins may be a fundamental resolution strategy for retarding cellular senescence in DN. Some researchers are focusing on the improvement of protein-bound uremic waste clearance through intravenous lipid emulsion [231] or infusion of a binding competitor ibuprofen [232] in conventional dialysis therapies. Although these strategies present benefits, they are still in the primary stage of research. Compared with the controlling in the clearance, to decrease the generation of uremic wastes through the modulation of beneficial gut microbiota members may be more important [233, 234]. It is proved in long-term safety and presents improved removal of BUN when probiotics is supplemented to the therapies for CKD patients [235]. Furthermore, synbiotics, resistant starch, and other dietary fiber could also help to decrease the production of uremic toxins through modifying gut microbial dysbiosis in CKD patients [236], thereby ameliorating oxidative stress and inflammation [237, 238]. However, the formulation of a probiotic bacteria and the detailed therapies in clinic need to be further studied, and the safety is also an important question to be resolved.

## 5. Conclusion

In summary, we show the multiple mechanisms underscoring cell senescence network in diabetic nephropathy, which involve telomere shortening, DNA damage, epigenetic alterations, mitophagy deficiency, loss of Klotho, Wnt/*β*-catenin signaling activation, inflammation, and the accumulation of uremic toxins. These factors mutually affect and cooperatively promote cellular senescence in DN. Although some therapeutic methods are promising, the practical application in DN patients needs to be testified by large amounts of animal experiments and preclinical investigations. Notably, the long-term observation of antiaging therapy such as senolytics and stem cell transplantation could not be neglected. Recently, genome editing is attracting the notice of scientists [239, 240]. However, although with the prospective aspects, genome editing would bring out the problems of ethics in humanity. This should be particularly focused. Last, precision medicine therapy for individual DN patient should be considered in the therapeutic strategies of nephrologists. Nevertheless, targeted inhibition of cellular senescence provides important clues for clinical strategies for diabetic nephropathy.
